# Fractional anisotropy of the uncinate fasciculus and cingulum in bipolar disorder type I, type II, unaffected siblings and healthy controls

**DOI:** 10.1192/bjp.2018.101

**Published:** 2018-09

**Authors:** Sonya F. Foley, Matthew Bracher-Smith, Katherine E. Tansey, Judith R. Harrison, Greg D. Parker, Xavier Caseras

**Affiliations:** 1 scientific support staff, Cardiff University Brain Research Imaging Centre, Cardiff University, UK; 2 PhD student, MRC Centre for Neuropsychiatric Genetics and Genomics, Institute of Psychological Medicine and Clinical Neurosciences, Cardiff University, UK; 3 Core Bioinformatics and Statistics Team, College of Biomedical and Life Sciences, Cardiff University, Cardiff, UK; 4 clinical research fellow, MRC Centre for Neuropsychiatric Genetics and Genomics, Institute of Psychological Medicine and Clinical Neurosciences, Cardiff University, UK; 5 senior data analyst, Cardiff University Brain Research Imaging Centre, Cardiff University, UK; 6 faculty member, MRC Centre for Neuropsychiatric Genetics and Genomics, Institute of Psychological Medicine and Clinical Neurosciences, Cardiff University, UK

**Keywords:** Bipolar disorder, DTI, fractional anisotropy, uncinate fasciculus, cingulum, polygenic score, ALSPAC

## Abstract

**Background:**

Fractional anisotropy in the uncinate fasciculus and the cingulum may be biomarkers for bipolar disorder and may even be distinctly affected in different subtypes of bipolar disorder, an area in need of further research.

**Aims:**

This study aims to establish if fractional anisotropy in the uncinate fasciculus and cingulum shows differences between healthy controls, patients with bipolar disorder type I (BD-I) and type II (BD-II), and their unaffected siblings.

**Method:**

Fractional anisotropy measures from the uncinate fasciculus, cingulum body and parahippocampal cingulum were compared with tractography methods in 40 healthy controls, 32 patients with BD-I, 34 patients with BD-II, 17 siblings of patients with BD-I and 14 siblings of patients with BD-II.

**Results:**

The main effects were found in both the right and left uncinate fasciculus, with patients with BD-I showing significantly lower fractional anisotropy than both patients with BD-II and healthy controls. Participants with BD-II did not differ from healthy controls. Siblings showed similar effects in the left uncinate fasciculus. In a subsequent complementary analysis, we investigated the association between fractional anisotropy in the uncinate fasciculus and polygenic risk for bipolar disorder and psychosis in a large cohort (*n* = 570) of healthy participants. However, we found no significant association.

**Conclusions:**

Fractional anisotropy in the uncinate fasciculus differs significantly between patients with BD-I and patients with BD-II and healthy controls. This supports the hypothesis of differences in the physiological sub-tract between bipolar disorder subtypes. Similar results were found in unaffected siblings, suggesting the potential for this biomarker to represent an endophenotype for BD-I. However, fractional anisotropy in the uncinate fasciculus seems unrelated to polygenic risk for bipolar disorder or psychosis.

**Declaration of interest:**

None.

Bipolar disorder can be subdivided into bipolar disorder type I (BD-I) and bipolar disorder type II (BD-II) based on the intensity, duration or presence of psychotic symptoms during episodes of ‘high’ mood.^1^ Therefore, BD-II has been regarded as a milder form of BD-I, with both subtypes sharing the same neurophysiological underpinnings; however, recent research has shown clear divergences in their clinical presentation and epidemiology^2^ and their genetic basis,^3^ suggestive of a more fundamental difference between subtypes. Taking both subtypes together, bipolar disorder is highly heritable and influenced by the additive effect of a large number of genes.^4^ Current theoretical models point at inefficient connectivity between prefrontal brain areas and subcortical structures associated with emotion and cognition as the basis of the extreme mood episodes present in bipolar disorder.^5^^–^^7^ This hypothesis finds support from several studies reporting differences in white matter microstructure measures between participants with bipolar disorder and healthy controls, as measured by magnetic resonance imaging (MRI).^8^^–^^13^ Among white matter tracts studied, the uncinate fasciculus and the cingulum bundle are suggested to be at the core of the emotion regulation circuitry,^12^^,^^14^ and several studies have reported reduced fractional anisotropy in both these tracts in participants with bipolar disorder compared with healthy controls.^7^^,^^9^^,^^15^^–^^17^ The few available studies comparing white matter microstructure between participants with BD-I and BD-II have shown differences in fractional anisotropy across subtypes.^18^^–^^20^ Our group^15^ has previously used tractography to focus on the uncinate fasciculus and showed reduced fractional anisotropy in participants with BD-I compared with participants with BD-II and healthy participants, along with behavioural and blood oxygenation level dependent (BOLD) responses to an emotion paradigm suggestive of emotion regulation deficits in participants with BD-I, but not in participants with BD-II. White matter microstructure in the uncinate fasciculus was thus posited as a potential biomarker, which could explain the phenotypic differences between bipolar disorder subtypes. Unaffected relatives of patients with bipolar disorder also have reduced fractional anisotropy^21^ in whole-brain analyses, supporting the idea of this representing a potential endophenotype for the disorder.

In this study, we aimed to confirm our previous results of reduced fractional anisotropy in the uncinate fasciculus of participants with BD-I compared with participants with BD-II and healthy controls by using a larger sample, and to extend this comparison into the cingulum bundle. We restricted our analyses to these tracts based on previous literature and the proposed role of these in emotion regulation because of their anatomical location. We also explored whether fractional anisotropy reductions in tracts showing differences between participants with bipolar disorder and healthy controls are also present in non-affected siblings of participants with bipolar disorder, indicative of a familial aggregation. Finally, we examined whether fractional anisotropy in these tracts is associated with polygenic risk for bipolar disorder^22^ and/or psychosis.^23^

## Method

### Participants

The total sample included 137 participants: 66 patients with bipolar disorder (32 with BD-I and 34 with BD-II), 31 of their unaffected siblings (17 siblings of participants with BD-I and 14 siblings of participants with BD-II) and 40 healthy controls. All patients were recruited from the National Centre for Mental Health (http://www.ncmh.info) and the Bipolar Disorder Research Network (http://bdrn.org). These were well-characterised patients previously diagnosed by trained researchers using standardised clinical interviews. A clinically trained and experienced researcher (X.C.) further interviewed participants with the Mini-International Neuropsychiatric Interview (MINI)^24^ to confirm diagnosis and suitability for inclusion. Unaffected siblings were contacted via recruited patients, and only one sibling was included from each family. Healthy controls were recruited from the community via advertisement. Both healthy controls and siblings were also interviewed with the MINI to verify suitability for inclusion. Participants were invited if they were aged >35 years to minimise risk of siblings and healthy controls developing bipolar disorder or psychosis in the future. All participants passed institutional MRI safety screening and had no history of neurological disorders or brain injuries. Participants with bipolar disorder had been euthymic – defined as ‘absence of significant mood episodes or changes in treatment received’ – for at least 2 months before scanning. Exclusion criteria for all participants included presence of alcohol/substance dependence within the past 12 months. To avoid confounding diagnosis with other psychotic syndromes (e.g. schizophrenia or schizoaffective), participants with bipolar disorder were excluded if they reported any positive history of delusions or hallucinations outside a mood episode. Unaffected siblings were excluded if they reported any personal history of mood disorder or psychosis. Healthy controls were excluded if they reported any personal history of any mental disorders or family history of bipolar disorder or psychosis in first-degree relatives.

All participants gave written informed consent before inclusion in the study and completed the Hamilton Depression Rating Scale (HDRS^25^), the Young Mania Rating Scale (YMRS^26^) and the National Adult Reading Test (NART^27^) on the day of the scan. The study was approved by the South East Wales Research Ethics Committee (REC ref: 08/WSE04/67).

### Polygenic risk analysis sample

Based on our results, a secondary analysis of fractional anisotropy in the uncinate fasciculus was performed looking separately at polygenic risk scores (PRSs) for bipolar disorder^22^ and psychosis^23^ in a subgroup of participants (*n* = 661; mean age 19.7 years; 81% male) from the Avon Longitudinal Study of Parents and Children (ALSPAC). ALSPAC is a population-based birth cohort of 14 062 live births – with expected delivery dates between 1 April 1991 and 31 December 1992 – of which 13 988 children were alive at 1 year of age. Data within ALSPAC has been reported extensively,^28^^,^^29^ and the study website contains details of all the data that is available through a fully searchable data dictionary (http://www.bristol.ac.uk/alspac/researchers/our-data/). Ethical approval for the study was obtained from the ALSPAC Ethics and Law Committee and the local National Health Service research ethics committee.

### MRI data acquisition

MRI imaging for both samples (case–control and ALSPAC) was performed on the same GE HDx 3T scanner (GE Healthcare, Milwaukee WI) at Cardiff University Brain Research Imaging Centre. A T1-weighted brain scan was acquired for co-registration by an axial three-dimensional fast spoiled gradient recalled sequence (repetition time (TR)/echo time (TE)/inversion time (TI) = 8/3/450 ms; flip angle 20°; acquisition matrix 256(anterior–posterior) × 192(left–right) × 172(superior–inferior), 1 mm isotropic voxels), followed by a diffusion tensor imaging (DTI) sequence with a twice-refocused spin-echo echo-planar parallel to the AC-PC plane. Acquisition was peripherally gated to the cardiac cycle. Data were obtained from 60 slices of 2.4 mm thickness (field of vision 230 mm, matrix size 96 × 96, TE = 87 ms and parallel imaging (Array coil Spatial Sensitivity Encoding Technique (ASSET) factor 2), b-values 0 and 1200 s/mm^2^), encoding diffusion along 30 isotropically distributed directions and three non-diffusion-weighted scans according to an optimised gradient vector scheme.^30^ Part of the ALSPAC sample (*n* = 219) was acquired as 60 directions, of which the optimal 30 directions were selected^31^ together with the first three b-value 0 images, to make this data-set equivalent to the above and to allow for joint processing of all data.

### DTI data processing

DTI data were processed with ExploreDTI version 4.8.3.^32^ First, T1 structural data were downsampled to 1.5 × 1.5 × 1.5 mm resolution. Eddy current and participant motion correction were performed with an affine registration to the non-diffusion-weighted images.^33^ Echo-planar imaging correction of the DTI data was performed, warping the data to the downsampled T1 three-dimensional fast spoiled gradient recalled sequence.^34^ RESTORE^35^ and RESDORE^36^ corrections were run, together with free water correction.^37^ Whole-brain tractography was performed with a damped Richardson–Lucy algorithm.^38^ Termination criteria were an angle threshold > 45°, fiber orientation density function peak < 0.05 and fractional anisotropy < 0.2.

For the case–control sample, fibre tracts were obtained through an automated tractography pipeline,^39^ informed by manual tractography performed by a researcher (S.F.F.). As our method of segmenting the cingulum does not include the majority of the parahippocampal part of the cingulum bundle (PHC), this was calculated separately. Each automatically reconstructed tract was visually inspected in ExploreDTI and edited where necessary, to reach the same quality as manual tractography. During this process, the researcher (S.F.F.) was kept blind to participant's group allocation. For an example of tracts see supplementary Appendix 1 available at https://doi.org/10.1192/bjp.2018.101. Where it was not possible to successfully reconstruct a tract, this was excluded. The final numbers included were left uncinate fasciculus, *n* = 131; right uncinate fasciculus, *n* = 137; right and left cingulum body, *n* = 137; left PHC, *n* = 135 and right PHC, *n* = 134.

For the ALSPAC PRS group, an automated tractography script model was used to segment the uncinate fasciculus. After validation of the automatic tractography model, tracts flagged up because of a small number of streamlines or low fractional anisotropy values were manually checked, and deleted when tracts were unsuccessfully reconstructed. Final numbers were *n* = 652 for the left uncinate fasciculus *n* = 658 for the right uncinate fasciculus.

In both cases, fractional anisotropy values were extracted for the tracts of interest, by calculating the average of fractional anisotropy measures at each vertex of each streamline in the tract.

### Statistical analysis

Demographic and clinical variables were compared across groups by analysis of variance (ANOVA), except for YMRS and HDRS scores, for which suitable non-parametric tests (Kruskal–Wallis) were used. Gender distribution across groups was compared by χ^2^ test. Clinical descriptors (not available in all participants) were compared between participants with BD-I and BD-II by *t*-tests. Fractional anisotropy was compared by means of two separated ANOVAs, one including BD-I, BD-II and healthy controls, and a second including siblings of participants with BD-I, siblings of participants with BD-II and healthy controls. In both cases, Fisher's least significant difference *post hoc* testing was applied where appropriate. Bonferroni correction was used to correct for multiple comparisons.

### Polygenic risk analysis

Quality-controlled genotype data were received from the University of Bristol. Briefly, a total of 9912 participants from the ALSPAC study were genotyped with the Illumina HumanHap550 quad genome-wide single nucleotide polymorphism (SNP) genotyping platform (Illumina Inc., San Diego, California, USA) by 23andMe, subcontracting the Wellcome Trust Sanger Institute (Cambridge, UK) and the Laboratory Corporation of America (Burlington, North Carolina, USA). Individuals were removed if they had undetermined X-chromosome heterozygosity; abnormal heterozygosity; cryptic relatedness up to third-degree relatives, using identity by descent; genotyping completeness < 97%; and non-European ethnicity admixture detected by a multidimensional scaling analysis seeded with HapMap2 individuals. Markers with minor allele frequency (MAF) < 0.01, complete genotyping < 95% and an exact test of Hardy–Weinberg equilibrium (*P* < 5E−07) were removed. After quality control, 8365 unrelated individuals and 500 527 genotyped SNPs were available for analysis. Autosomal chromosomes were imputed with the reference panel HRCv1.1 (hrc.r1.1.2016),^40^ using a mixed population panel. Phasing was done by Eagle v2.3^41^ and imputation was done by Mimimac3.^42^ Imputed data were converted to best guess genotypes, using plink 1.9^43^ with multiallelic sites, and variants with an exact test of Hardy–Weinberg equilibrium (*P* < 1E−06) and MAF < 0.01 were removed.

PRSs were calculated according to the International Schizophrenia Consortium method.^22^ Two polygenic scores were generated: one for bipolar disorder^22^ and one for bipolar disorder plus schizophrenia cases versus healthy controls.^23^ which can be considered a proxy for psychosis, and can therefore be more predictive of BD-I than BD-II in our sample.^44^ Training data for bipolar disorder were taken from the Cross-Disorder Group of the Psychiatric Genomics Consortium,^45^ with 6990 individuals with bipolar disorder and 4820 controls. For combined bipolar disorder and schizophrenia score, data from Ruderfer *et al*^23^ on 19 779 individuals (10 410 with bipolar disorder and 9369 with schizophrenia) and 19 423 non-overlapping controls were used as training data. Scores were generated in plink^43^ (with --score), using six nested progressive *P*-value thresholds of 0.00001, 0.0001, 0.01, 0.1, 0.3 and 0.5. SNPs with MAF < 0.1 and imputation quality < 0.9 were removed. Linkage-disequilibrium independent SNPs were retained by informative pruning in plink^43^ (--clump to remove SNPs with linkage disequilibrium  > 0.1). A total of 570 individuals had both PRSs and brain imaging phenotypes available. Linear regression was performed separately for each PRS threshold and left and right fractional anisotropy values, using PRS as the explanatory variable and adjusting for age and gender as covariates.

## Results

### Demographics and clinical description

Groups did not differ with regards to age, gender distribution or performance on the NART (*P* > 0.1). As expected, both bipolar disorder groups showed higher scores in the HDRS and YMRS than the other groups (both *P* < 0.01), although scores remained well below clinical thresholds (Table 1).

**Table 1:** Demographic characteristics and psychometric scores across groups

	Healthy controls *n* = 40	BD-I *n* = 32	BD-II *n* = 34	Sib-I *n* = 17	Sib-II *n* = 14	Group comparison
Females, *n* (%)	24 (60)	22 (68)	19 (56)	9 (53)	9 (64)	χ^2^ (4) = 1.710, *P* > 0.1
Age, mean (s.d.)	43.5 (4.9)	45.0 (6.2)	42.8 (7.0)	47.2 (5.5)	43.6 (8.0)	F(4,132) = 1.763, *P* > 0.1
NART^a^, mean (s.d.)	36.8 (7.0)	35.2 (8.3)	34.8 (6.9)	36.1 (5.5)	35.4 (7.9)	F(4,121) = 0.348, *P* > 0.1
YMRS, mean (s.d.)	0.45 (0.8)	2.72 (2.4)	2.65 (3.0)	0.53 (0.7)	0.86 (1.1)	χ^2^ (4) = 46.885, *P* < 0.01
HDRS, mean (s.d.)	0.48 (0.9)	3.50 (3.1)	3.85 (3.7)	1.00 (1.2)	1.57 (1.9)	χ^2^ (4) = 26.854, *P* < 0.01

BD-I, participants with bipolar disorder type I; BD-II, participants with bipolar disorder type II; HDRS, Hamilton Depression Rating Scale; NART, National Adult Reading Test; Sib-I, unaffected siblings of participants with bipolar type I; Sib-II, unaffected siblings of participants with bipolar type II; YMRS: Young Mania Rating Scale.

a Number of correct responses in the NART is reported.

On average, participants with bipolar disorder in our study experienced their first mood episode at age 19 years despite first being diagnosed with bipolar disorder at age 31 years; those variables did not differ between BD-I and BD-II groups (t(50) = 0.21, *P* > 0.1; and t(54) = 1.24, *P* > 0.1, respectively). Only nine participants in our clinical sample (14%) were free of medication at the time of this study, whereas the majority (*n* = 36, 65%) were taking a combination of at least two different class of drugs (see Table 2 for details). One-third of the participants with bipolar disorder (*n* = 22; 12 with BD-I, 10 with BD-II) had history of at least one anxiety-related comorbid diagnosis, with panic disorder with/without agoraphobia being the most prevalent (31% of participants with BD-I, 35% of participants with BD-II), followed by obsessive–compulsive disorder (13% of participants with BD-I, 12% of participants with BD-II), with health anxiety, eating disorder and generalised anxiety disorder present at a much lower rate (3% of the total sample, *n* = 2). Because of the strict inclusion criteria, no other diagnoses were present in this sample. Only two siblings presented with mental health history, referring past history of generalised anxiety disorder (*n* = 1) and health anxiety (*n* = 1). Following our inclusion criteria, control participants had no history of any psychiatric disorder.

**Table 2:** Clinical characterisation of participants with BD-I and BD-I

	BD-I *n* = 32	BD-II *n* = 34	Group comparison
Age at first episode, mean (s.d.)	19 (5.8)	19 (7.0)	t (50) = 0.21, *P* > 0.1
Age at diagnosis, mean (s.d.)	30 (6.9)	34 (9.5)	t (54) = 1.24, *P* > 0.1
Co-diagnosis of anxiety disorder, *n* (%)	12 (38)	10 (29)	χ^2^ (1) = 0.49, *P* > 0.1
Medication, *n* (%)^a^			
Non-medicated	1 (3)	8 (24)	χ^2^ (1) = 5.60, *P* < 0.05
Antidepressants	16 (50)	15 (44)	χ^2^ (1) = 3.65, *P* > 0.1
Lithium	8 (25)	7 (21)	χ^2^ (1) = 2.49, *P* > 0.1
Anticonvulsants	16 (50)	15 (44)	χ^2^ (1) = 3.65, *P* > 0.1
Antipsychotics	20 (63)	7 (21)	χ^2^ (1) = 12.88, *P* < 0.001
Combination of above	22 (69)	14 (41)	χ^2^ (1) = 5.82, *P* < 0.05

BD-I, participants with bipolar disorder type I; BD-II, participants with bipolar disorder type II.

a Medication data from one participant with BD-I was inaccessible.

### Fractional anisotropy between-groups analyses

**Fig. 1:**
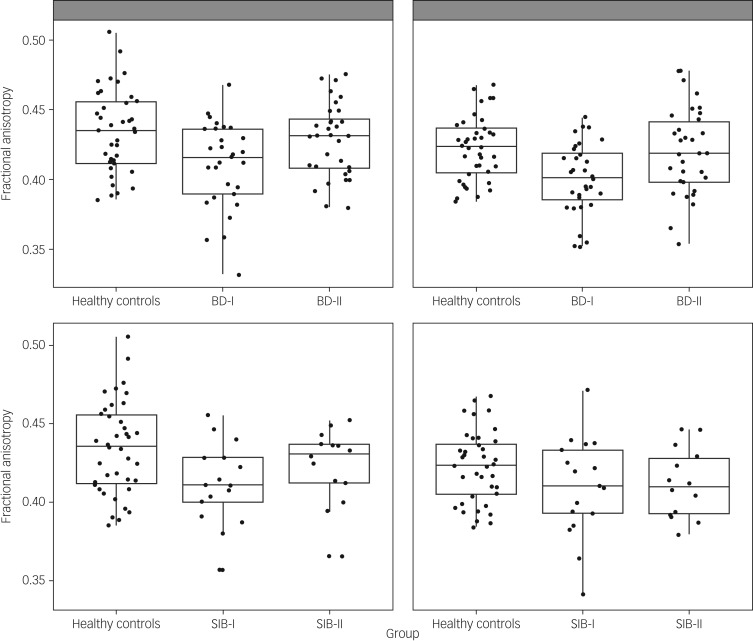
Scatterplot presenting fractional anisotropy values across healthy controls and participants with bipolar disorder type I (BD-I), type II (BD-II) (top panel), and unaffected siblings of bipolar type I (SIB-I) and type II (SIB-II) participants (bottom panel). Figures on the left correspond to the left uncinate fasciculus, figures on the right to the right uncinate fasciculus.

ANOVAs comparing fractional anisotropy across patients and controls showed a significant group effect in left and right uncinate fasciculus (F(2,97) = 5.87, *P* = 0.004; F(2,103) = 7.22, *P* = 0.001; respectively). These comparisons survived Bonferroni correction. *Post hoc* analysis showed that in both cases the effect was driven by participants with BD-I showing reduced fractional anisotropy compared with both healthy controls (*P* = 0.001 for both left and right) and participants with BD-II (*P* = 0.019 and *P* = 0.003, respectively, for left and right). However, there was no difference between participants with BD-II and healthy controls (*P* > 0.1) (Fig. 1). To exclude an effect of age in these results, the analyses were repeated with age as a covariate, and results remained almost identical.

ANOVAs including fractional anisotropy in cingulum body or PHC showed no significant group effect (left cingulum body F(2,103) = 2.53, *P* = 0.08; right cingulum body F(2,103) = 1.37; left PHC F(2,101) = 0.011; right PHC F(2,101) = 1.30; all other *P* > 0.1). Mean fractional anisotropy and s.d. for all tracts across groups are presented in supplementary Appendix 2.

Because this study contained a number of participants (BD-I *n* = 15, BD-II *n* = 14 and healthy controls *n* = 18) included in our previously reported results,^15^ we re-ran the tests with only newly recruited participants. Results mainly replicated those listed above, with both left and right uncinate fasciculus being significantly different between groups, a result which survived Bonferroni correction. *Post hoc* testing again showed fractional anisotropy of participants with BD-I to be less than that of healthy controls (supplementary Appendix 3).

Based on the results above, we ran ANOVAs for fractional anisotropy in left and right uncinate fasciculus including unaffected siblings and healthy controls. Results showed a significant group effect in the left uncinate fasciculus (F(2,67) = 3.47, *P* = 0.037) but not in the right uncinate fasciculus (F(2,68) = 2.03, *P* > 0.1). *Post hoc* pairwise comparisons showed that the left uncinate fasciculus effect was again driven by siblings of participants with BD-I showing reduced fractional anisotropy compared with healthy controls (*P* = 0.012). However, in this case, no other *post hoc* comparison was significant, and the main ANOVA's group effect did not survive Bonferroni correction. For completeness, we ran the ANOVAs for fractional anisotropy in the cingulum body and PHC, but again, none of these resulted in a significant group effect: left cingulum body F(2,68) = 0.38; right cingulum body F(2,68) = 1.35; left PHC F(2,68) = 0.32; right PHC F(2,67) = 1.65; all *P* > 0.1.

Finally, *post hoc* paired sample *t*-tests comparing participants with bipolar disorder and their unaffected siblings showed no significant difference in the uncinate fasciculus between those with BD-I and siblings of participants with BD-I (left: t(12) = 0.172, *P* > 0.1; right: t(13) = 1.286, *P* > 0.1) or those with BD-II and siblings of participants with BD-II (left: t(10) = 0.448, *P* > 0.01; right: t(10) = 0.784, *P* > 0.1).

### Medication effects on BD-I and BD-II

Point-biserial correlations between each medication class status (i.e. taking versus not taking) and fractional anisotropy in the uncinate fasciculus showed a significant positive association between lithium status and fractional anisotropy in the left uncinate fasciculus (*r* = 0.37, *P* = 0.004) and negative association between anticonvulsant status and right uncinate fasciculus (*r* = −0.26, *P* = 0.036). There were trends between lithium status and fractional anisotropy in the right uncinate fasciculus (*r* = 0.24, *P* = 0.055), and combination of drugs treatment status and fractional anisotropy in the left uncinate fasciculus (*r* = 0.24, *P* = 0.069). No other association approached significance. Regression analyses were then run to ascertain whether clinical group allocation (i.e. BD-I versus BD-II) still explained a significant amount of variance after accounting for fractional anisotropy variance explained by those medication variables. Clinical group allocation (BD-I versus BD-II) still explained a significant amount of fractional anisotropy in the left uncinate fasciculus after accounting for variance explained by lithium status (*R*^2^ change 0.103, F(1,57) = 7.73, *P* = 0.007), and the same was found for the right uncinate fasciculus (*R*^2^ change 0.124, F(1,62) = 9.42, *P* = 0.003). Similarly, clinical group significantly predicted fractional anisotropy in the right uncinate fasciculus after accounting for variance associated with anticonvulsant status (*R*^2^ change 0.101, F(1,62) = 7.57, *P* = 0.008), and in the left uncinate fasciculus after accounting for variance explained by combined drugs treatment (*R*^2^ change 0.149, F(1,57) = 10.72, *P* = 0.002).

### Association between fractional anisotropy in the uncinate fasciculus and polygenic risk for bipolar disorder and psychosis

There was no significant correlation between polygenic risk for bipolar disorder or psychosis at any of the six *P*-value thresholds used and fractional anisotropy in the left or right uncinate fasciculus. In all cases, *R*^2^ values remained very low (<0.01; supplementary Appendix 4).

## Discussion

This study contributes to the gradual unravelling of the neuropathophysiology of bipolar disorder. Our main aim was to test our hypothesis of reduced fractional anisotropy in the uncinate fasciculus in patients with BD-I, but not BD-II. We also aimed to extend this research into the cingulum, where we expected the same pattern of effects. The results supported the former, but not the latter. Interestingly, white matter microstructure differences in the uncinate fasciculus were partially mirrored in unaffected siblings, suggesting this as a potential endophenotype specifically for BD-I. Despite the potential genetic basis of this difference suggested by our results in siblings, we did not find any association between fractional anisotropy in the uncinate fasciculus and polygenic scoring for bipolar disorder or psychosis in a larger, independent population sample.

Our results confirmed previously reported findings of decreased fractional anisotropy in the uncinate fasciculus of participants with bipolar disorder.^10^^,^^11^^,^^19^^,^^46^ We also confirmed previous findings from our group^15^ in a larger sample, showing this to be the case for BD-I, but not BD-II. Rather than showing an intermediate position between BD-I and healthy controls, fractional anisotropy in the uncinate fasciculus for BD-II behaved indistinguishably from healthy controls, but significantly differed from BD-I. This result indicates a potential distinct pathophysiological mechanism between bipolar subtypes, rather than supporting the hypothesis of a simple difference in symptom severity, and merits further investigation. Interestingly, previous research has found a similar pattern of white matter deficits in frontal and parietal regions between patients with BD-I and schizophrenia.^46^ In accordance with the diagnostic criteria, the patients recruited for this study with a BD-II diagnosis did not experience psychosis during episodes of high mood and in our sample, most people with BD-II also denied experiencing psychotic symptoms during depression. The majority of our participants with BD-I did report experiencing psychotic symptoms during mania. Thus, this difference in fractional anisotropy between bipolar disorder subtypes in the uncinate fasciculus could reflect vulnerability to psychosis rather than mood symptoms. Future research should address this question, looking at fractional anisotropy in the uncinate fasciculus as a potential crosscutting biomarker for psychosis following the research domain criteria perspective.^47^

Our results did not concur with previous findings in the cingulum indicating reduced fractional anisotropy in bipolar disorder compared with healthy controls,^9^^,^^12^^,^^19^^,^^20^ even when combining all participants with bipolar disorder into a single group (supplementary Appendix 5). This could be explained by methodological differences. Most previous findings for the cingulum were based on voxel-wise comparisons^8^^,^^19^^,^^20^ or used a very different tract mapping methodology^9^^,^^12^ than in this study. To our knowledge, only two previous studies used equivalent methods of fibre tracking to this study,^10^^,^^13^ one of which also failed to observe significant group effects for the cingulum.^10^ The advantage of tractography over voxel-wise methods is its power to detect effects in predefined tracts of interest rather than representing an exploratory approach across the whole brain, requiring more correction for multiple comparisons. Because of our strong *a priori* hypotheses about the uncinate fasciculus, cingulum body and PHC, tractography was selected as the most adequate approach. In any case, our results should call into question the relevance of fractional anisotropy in the cingulum as a potential biomarker for bipolar disorder.

Interestingly, our results are also suggestive of reduced fractional anisotropy in the left uncinate fasciculus for siblings of participants with BD-I compared with healthy controls, but not for siblings of participants with BD-II. No significant group effect was found in the right uncinate fasciculus despite *post hoc* testing showing a trend toward lower fractional anisotropy, again between siblings of participants with BD-I and healthy controls (*P* = 0.08). We also found that both sibling groups did not differ from their affected relatives with regards to fractional anisotropy in the uncinate fasciculus. These findings are consistent with previous research reporting siblings of patients with bipolar disorder showing intermediate fractional anisotropy values between patients and controls in various white matter regions,^48^ and therefore placing fractional anisotropy in the uncinate fasciculus as a potential endophenotype for this disorder. This is the first time that this result incorporating patients and their siblings has been reported based on tractography on *a priori* selected tracts of interest rather than on a whole-brain voxel-wise exploratory approach. Importantly, our sibling groups were selected on the basis of an absence of any personal history of mood disorders and psychosis, and their age (>35 years), which indicates lower probability of developing bipolar disorder in the future;^49^ as a consequence, our sibling groups can be considered at a higher familial risk but yet resilient. This strengthens the hypothesis of lower fractional anisotropy in the uncinate fasciculus representing an endophenotype for BD-I, likely to be present as a premorbid risk marker independent of mental state and psychopathological history, and endorses future research in this area.

Considering the results discussed so far and previous literature showing fractional anisotropy to be heritable,^50^ one could have predicted that common genetic variance associated with bipolar disorder or psychosis would correlate with fractional anisotropy in the uncinate fasciculus, which surprisingly was not the case. Among currently available genome-wide association studies for bipolar disorder, BD-I is over-represented compared with BD-II,^51^ and therefore we predicted that any bipolar disorder polygenic score based on this data would mainly represent BD-I risk. Also, as we advocated earlier, if fractional anisotropy in the uncinate fasciculus reflects vulnerability for psychosis rather than mood disorder, one would expect the polygenic score for psychosis^23^ to also correlate with fractional anisotropy in the uncinate fasciculus. However, our results did not confirm this notion, calling into question whether the familial effects reported above are driven by common genetic factors. Further replication in larger samples and with more suited polygenic scores than the ones currently available (i.e. specific for BD- I or BD-II) would be required before any firm conclusions can be drawn.

Our study has some limitations that should be noted. First our bipolar disorder groups include participants used in a previous study reporting fractional anisotropy differences in uncinate fasciculus.^15^ It is important to notice, however, that these two samples were acquired in the same MRI machine and with exactly the same acquisition sequence, and all data were (re)processed together for this study. We also replicated our analyses after excluding those participants who took part in the previous study, and obtained very similar results, albeit with decreased statistical power (supplementary Appendix 3). Second, because of our stringent inclusion criteria (i.e. lack of any history of mood disorder or psychosis, age >35 years and only one sibling per family), our sample size for unaffected siblings is modest, which has limited our power to detect significant effects. Despite the limitation in sample size, the use of such a group of siblings strengthens our conclusion regarding fractional anisotropy in the uncinate fasciculus as a potential endophenotype for BD-I. Finally, the potential confounding effect of medication is a common problem in studies recruiting patients with chronic illnesses like bipolar disorder. Despite this, we have shown that differences in fractional anisotropy in the uncinate fasciculus found between participants with BD-I and BD-II were not fully explained by medication status. Moreover, it has been suggested that medication effects are smaller than originally thought but could increase type II errors because of the normalisation effect over brain function and structure.^52^ With this in mind, we believe that our main results regarding differences between patients and controls are not secondary to medication.

In conclusion, we showed the microstructure of white matter in the uncinate fasciculus to be compromised in individuals with BD-I but not in individuals with BD-II, compared with healthy controls. A similar effect, albeit reduced, was seen in unaffected siblings of participants with BD-I, indicating familiality and postulating fractional anisotropy in the uncinate fasciculus as a potential endophenotype for bipolar disorder.

## Supplementary material

For supplementary material accompanying this paper visit https://doi.org/10.1192/bjp.2018.101. click here to view supplementary material
