# Supplementary material for: Fractional anisotropy of the uncinate fasciculus and cingulum in bipolar disorder type I, type II, unaffected siblings and healthy controls
Source: Br J Psychiatry. 2018 Sep;213(3):548–54. doi: 10.1192/bjp.2018.101 (PMC6130806; doi:10.1192/bjp.2018.101)
Supplement: Supplementary file 1 [file S0007125018001010sup001.docx]

**Appendix 1 – Illustration of Tractography**

fx1

**Figure Appendix 1:** Example of tracts obtained, overlaid on DTI map. Red = uncinate fasciculus, green = cingulum body, blue = parahippocampal cingulum.

-------------------------------------------------------------------------------------------------------

**Appendix 2 – Mean fractional anisotropy (s.d.) for each tract of interest across groups**

|  | HC *n*= 40 | BD-I *n*= 32 | BD-II *n*= 34 | Sib-I *n*= 17 | Sib-II *n*= 14 |
| --- | --- | --- | --- | --- | --- |
| UF Left | 0.434 (0.030) | 0.410 (0.031) | 0.428 (0.026) | 0.413 (0.026) | 0.423 (0.024) |
| UF Right | 0.422 (0.023) | 0.400 (0.025) | 0.420 (0.031) | 0.410 (0.032) | 0.412 (0.022) |
| Cingulum Left | 0.434 (0.028) | 0.422 (0.033) | 0.439 (0.036) | 0.432 (0.044) | 0.426 (0.020) |
| Cingulum Right | 0.414 (0.025) | 0.403 (0.034) | 0.413 (0.028) | 0.425 (0.035) | 0.412 (0.019) |
| PHC Left | 0.357 (0.026) | 0.356 (0.027) | 0.356 (0.027) | 0.358 (0.029) | 0.363 (0.014) |
| PHC Right | 0.374 (0.035) | 0.384 (0.033) | 0.385 (0.031) | 0.391 (0.035) | 0.377 (0.018) |

UF: uncinate fasciculus; PHC: parahippocampal cingulum; HC: Healthy Controls; BD-I: bipolar disorder type I; BD-II: bipolar disorder type II; Sib-I: unaffected siblings of bipolar type I participants; Sib-II: unaffected siblings of bipolar type II participants.

-------------------------------------------------------------------------------------------------------

**Appendix 3 – ANOVA for FA in the uncinate fasciculi restricted to newly recruited bipolar and healthy control participants. FA differences are preserved in the right and left UF.**

Left uncinate F(2,53) = 4.105, *P*= 0.022; Right uncinate F(2,56) = 4.546, *P*= 0.015).

Table A3.1. Mean fractional anisotropy (s.d.) in the uncinate fasciculus across bipolar groups and healthy comparison participants

|  | HC *n*= 22 | BD-I *n*= 17 | BD-II *n*= 20 |
| --- | --- | --- | --- |
| Left uncinate | 0.440 (0.31) | 0.414 (0.034) | 0.422 (0.022) |
| Right uncinate | 0.421 (0.019) | 0.398 (0.025) | 0.415 (0.028) |

*Reconstruction of the left uncinate fasciculus failed for 2 BD-I and 1 BD-II participants and were consequently excluded from the analyses.

Table A3.2. *P* values resulting from post hoc pairwise comparisons (*LSD*) for FA in left and right uncinate fasciculus.

|  | Left uncinate | Right uncinate |
| --- | --- | --- |
| HC *v.* BD I | *0.009* | *0.004* |
| HC *v.* BD II | *0.049* | 0.388 |
| BD I *v.* BD II | 0.427 | *0.041* |

-------------------------------------------------------------------------------------------------------

**Appendix 4 - Bar graph of polygenic risk R2 values for left and right uncinate fasciculus fractional anisotropy (FA)**

fx2

**Figure** **Appendix 4**: Bar graph of polygenic risk R2 values for left and right uncinate fasciculus fractional anisotropy (FA) for the 6 *P*-value thresholds (PT): 0.00001, 0.0001, 0.01, 0.1, 0.3 and 0.5. **A**: polygenic risk for bipolar disorder, **B**: polygenic risk for psychosis.

-------------------------------------------------------------------------------------------------------

**Appendix 5 – ANOVA for FA in the cingulum body (CB) and parahippocampal cingulum (PHC) adding BD-I and BD-II participants together. No differences in FA were found.**

Left CB F(1,104) = 1.070, *P*= 0.303; Right CB F(1,104) = 2.456, *P*= 0.120).

Left PHC F(1,102) = 0.002, *P*= 0.962; Right PHC F(1,102) = 1.128, *P*= 0.291).

Table A5. Mean fractional anisotropy (s.d.) in the CB and PHC across bipolar and healthy comparison participants

|  | HC *n*= 40 | BD *n*= 66 |
| --- | --- | --- |
| Left CB | 0.434 (0.028) | 0.431 (0.035) |
| Right CB | 0.414 (0.025) | 0.408 (0.031) |
| Left PHC | 0.357 (0.026) | 0.356 (0.027) |
| Right PHC | 0.374 (0.035) | 0.385 (0.032) |

-------------------------------------------------------------------------------------------------------
